# Soluble epidermal growth factor receptor (sEGFR) and carcinoembryonic antigen (CEA) concentration in patients with non-small cell lung cancer: correlation with survival after erlotinib and gefitinib treatment

**DOI:** 10.3332/ecancer.2010.178

**Published:** 2010-11-03

**Authors:** I Kappers, M A Vollebergh, H van Tinteren, C M Korse, L L Nieuwenhuis, J M G Bonfrer, H M Klomp, N van Zandwijk, M M van den Heuvel

**Affiliations:** 1 Department of Surgery; 2 Department of Thoracic Oncology; 3 Department of Statistics; 4 Department of Clinical Chemistry, The Netherlands Cancer Institute–Antoni van Leeuwenhoek Hospital, Plesmanlaan 121, 1066 CX Amsterdam, The Netherlands

## Abstract

**Background:**

In patients with non-small cell lung cancer (NSCLC), a higher response rate can be achieved with epidermal growth factor receptor-tyrosine kinase inhibitors (EGFR-TKIs) when selection for therapy is guided by mutation analysis or gene amplification. However, both tests are complex and require tumour tissue. Simple methods to identify responders prior to EGFR-TKI treatment are urgently needed. This study aimed to define the relation between serum sEGFR levels, carcinoembryonic antigen (CEA) and survival in NSCLC patients treated with EGFR-TKIs.

**Methods:**

Patients with stage III/IV NSCLC treated with gefitinib or erlotinib between July 2002 and December 2005 were reviewed. Levels of serum soluble EGFR (sEGFR) were determined by a sandwich quantitative enzyme-linked immunosorbent assay. A chemiluminescence immunoassay was used for CEA. The relation between sEGFR and survival was investigated.

**Results:**

One hundred and two NSCLC patients, mainly stage IV (80%), were identified. Mean sEGFR at baseline was 55.9 μg/l (range 35.3–74.5 μg/l). The median CEA level was 11.1 μg/l (range <1.0–2938.0 μg/l). Median overall survival was 5.2 months (range 1–52 months). Decreasing log CEA values (HR 1.51, 95% CI 1.11–2.04, multivariate analysis) and increasing sEGFR values (HR 0.96, 95% CI 0.93–0.99, multivariate analysis) were both independently associated with prolonged survival. Higher levels of pre-treatment sEGFR were associated with lower risk of progressive disease within three months (*p*=0.04).

**Conclusions:**

Both baseline sEGFR and CEA levels in NSCLC patients receiving EGFR-TKIs showed a significant correlation with survival. To distinguish whether these factors have a predictive or a prognostic value, validation is warranted in an independent patient series containing a control arm without EGFR-TKI treatment.

## Background

The epidermal growth factor receptor (EGFR) is a receptor tyrosine kinase that is abnormally activated in different types of epithelial malignancies. A constitutively activated EGFR can lead to malignant transformation of the cell. It was shown 20 years ago that blocking of the EGFR could inhibit cell proliferation in these transformed cells [1]. Since these first observations various drugs have been developed that target either the extra-cellular domain or the intracellular tyrosine kinase domain of the EGFR. Especially, drugs of the latter category, small molecule adenosine triphosphate-competitive inhibitors of the receptor’s tyrosine kinase (EGFR-TKIs), such as erlotinib and gefitinib, have proven their efficacy in the treatment of non-small cell lung cancer (NSCLC) [2–4]. However, response rates of erlotinib and gefitinib in unselected patient populations are low, and selection of patients is warranted to increase response rates to a more satisfying level. A response rate of 30% can be achieved when selection of patients is based on their phenotype (female gender, non-smoking status, Asian origin, adeno- or bronchioloalveolar carcinoma) [5–9]. This can be increased up to 70% when selection is based on EGFR mutation or FISH analysis [10–18]. However, for these assays availability of tumour tissue is a prerequisite, while this is frequently not at hand in advanced NSCLC. More simple and accessible predictors of response are warranted.

Recently, soluble EGFR (sEGFR) was recognized as a potential screening tool for epithelial cancer [19,20]. SEGFR is a proteolytically cleaved form of the extra-cellular domain of the EGFR and can be measured directly in the serum [21,22]. The plausibility of sEGFR being a surrogate marker for response to treatment with an EGFR-TKI is based on the hypothesis that the level of sEGFR reflects the absolute number of activated receptors, susceptible to inhibition [23]. A decrease in sEGFR during treatment with gefitinib has been recognized to correlate with disease control in patients with NSCLC [24]. However, the role of baseline sEGFR as a predictive marker for response and survival in clinical practice is still uncertain.

The conventional tumour marker carcinoembryogenic antigen (CEA) is a member of the immunoglobulin supergene family, a cell surface adhesion protein, and it is thought to play a role in cell-to-cell adhesion(22). Since there is evidence that elevated pre-treatment levels of CEA are also predictive for response and outcome after the EGFR-TKI treatment, independent of histological subtype, we decided to study both baseline sEGFR and CEA levels in relation to survival after the EGFR-TKI treatment [25].

## Patients and methods

### Patients selection and study design

Between July 2002 and December 2005, patients with advanced non-small cell lung cancer, not responding to conventional chemotherapy or unable to receive chemotherapy due to poor medical condition, were offered treatment with gefitinib (Iressa®) or erlotinib (Tarceva®) as part of the Expanded Access Programme, on a compassionate use basis. Consecutive patients who were treated for more than 14 days were identified and enrolled in this study if pre-treatment serum was available for sEGFR analysis. The final sample size was determined according to the number of available patient serum samples. Hospital records were retrospectively reviewed for age, gender, race, smoking status, histological subtype, stage, side effects and toxicity of the EGFR-TKI treatment and best overall response to EGFR-TKI. Informed consent was obtained from all patients. For design and report of this study, the REMARK guidelines were followed [26].

Patients receiving gefitinib were treated with a daily dose of 250 mg. In case of unacceptable or severe (grades 3–4) toxicity, the treatment with gefitinib was interrupted. Erlotinib was administered in daily doses of 150 mg. Dose changes of 50 mg were possible in case of unacceptable toxicity. Adverse events were assessed according to the National Cancer Institute—Common Toxicity Criteria version 2. Treatment of gefitinib or erlotinib was continued until disease progression or the occurrence of a serious adverse event.

### Assessment of sEGFR and CEA levels

Blood samples had to be collected within two months before start of treatment with EGFR-TKIs. Serum was stored at −30°C. Concentration levels of the EGFR-extra-cellular-binding domain were determined by a sandwich quantitative enzyme-linked immunosorbent assay (EGFR Microtiter ELISA; Oncogene Science, Cambridge, MA) according to the manufacturer’s instructions. The normal range is 45–78 μg/l as described previously [27]. Carcinoembryonic antigen was measured on the E170 analyzer, which is based on chemiluminescent immunometric technology (Roche Diagnostics, Mannheim, Germany) [28].

### Response assessment and statistical analysis

Correlation among sEGFR, CEA and age were studied using Pearson correlation analysis. Associations between sEGFR, CEA, gender, stage (III, IV), smoking status (smoker, non-smoker) and histology (adenocarcinoma, squamous cell carcinoma and large cell undifferentiated) were investigated by means of the Student’s *t* test or generalized linear regression.

Overall survival was calculated using the Kaplan–Meier method, from the first day of treatment with the EGFR-TKI to the date of death. Differences in survival between subgroups of patients were determined using the log rank test. Univariate analysis (Cox proportional hazard regression analysis) was used to detect associations between sEGFR and CEA levels and survival. Furthermore, age, gender, smoking status, tumour stage, histology and treatment drug were investigated. The assumptions of linearity and proportional hazards for sEGFR and CEA were checked by means of Martingale residuals and scaled Schoenfeld residuals [29,30]. Continuous variables (age, sEGFR and logCEA) were tested for possible non-linear associations (violence of the proportional hazards assumptions). To present Kaplan–Meier plots for sEGFR and logCEA, a cut-off was used to divide these factors into two separate groups (i.e. high vs low). A spline function through the Martingale residuals of sEGFR and logCEA was used to determine possible cut-off values, i.e., the concentration of sEGFR or CEA, where the line crossed through zero of the Martingale residuals, was used as the cut-off. Variables achieving a probability value of less than 0.10 in the univariate analysis as well as pre-operative factors considered relevant in the available literature [31–33] were introduced in a multivariate stepwise proportional hazard analysis to identify variables significantly associated with survival. *p*-values < 0.05 were considered statistically significant.

Response evaluation was performed using computed tomography (CT) according to the Response Evaluation Criteria In Solid Tumors (RECIST) [34]. Response measurement at fixed intervals was not available for every patient. The occurrence of early progressive disease (PD) (within three months) was investigated to analyze the relation between (non-) response and sEGFR and/or CEA levels. Associations between high or low sEGFR and/or CEA levels, and early occurrence of PD were tested using non-parametric tests. For this purpose, sEFGR and log CEA were dichotomized by the cut-off value described above.

## Results

Over a 3.5 years period, 145 patients with advanced non-small cell lung cancer were treated with gefitinib or erlotinib. Of these, 102 patients with available serum samples were eligible, 54 men and 48 women, with a mean age of 59 years (95% CI 57–61 years). Patients’ characteristics are shown in Table 1. The median follow-up was 161 days (range 17–1581 days). EGFR mutation status was assessed in 13 patients, of whom six patients had mutations, three patients had a mutation in exon 19, one patient in exon 20 and two patients in exon 21. Sixty-seven patients were treated with gefitinib and 35 patients were treated with erlotinib. The median duration of treatment with gefitinib was 69 days (range 14–1259 days) and with erlotinib 78 days (range 15–814 days).

Baseline sEGFR levels were available for all 102 patients and showed a Gaussian distribution. The mean sEGFR level at baseline was 55.9 μg/l (SD 8.9). Given the normal range provided by the manufacturer of the test (48–72 μg/l), 23% of patients had decreased sEGFR levels. Patients with a squamous cell tumour had significant lower values of sEGFR compared to patients with tumours of the undifferentiated cell type (*p*=0.0267); sEGFR levels of patients with adenocarcinoma were found in between. Age was the only patient’s characteristic that significantly inversely correlated with sEGFR (correlation −0.31, *p*=0.0014). No significant associations were detected for sEGFR levels with gender, smoking status or tumour stage.

Baseline CEA values were available for 100 patients. CEA values did not follow a normal distribution. The median serum CEA value overall was 11.1 μg/l (range <1.0–2938.0 μg/l). Using the internationally accepted upper limit of normal of 6.5 μg/l for smokers and 5.0 μg/l for non-smokers, 67 patients (67%) had elevated CEA levels. Because of the skewed distribution of CEA, further analyses were performed using the logarithm of CEA (log CEA, mean 1.17, SD 0.75). Log CEA levels were significantly lower for stage III patients (*p*=0.01197) and for squamous cell compared to undifferentiated large cell type (*p*=0.0359). Values of patients with adenocarcinoma were very close to values of patients with tumours of the undifferentiated large cell type. For age, gender or level of sEGFR no association with log CEA was found.

When continuous variables (age, sEGFR and logCEA) were checked for possible non-linear associations, none were found to be significant. Consequently, the continuous variables could be included as linear continuous parameters.

**Figure 1:**
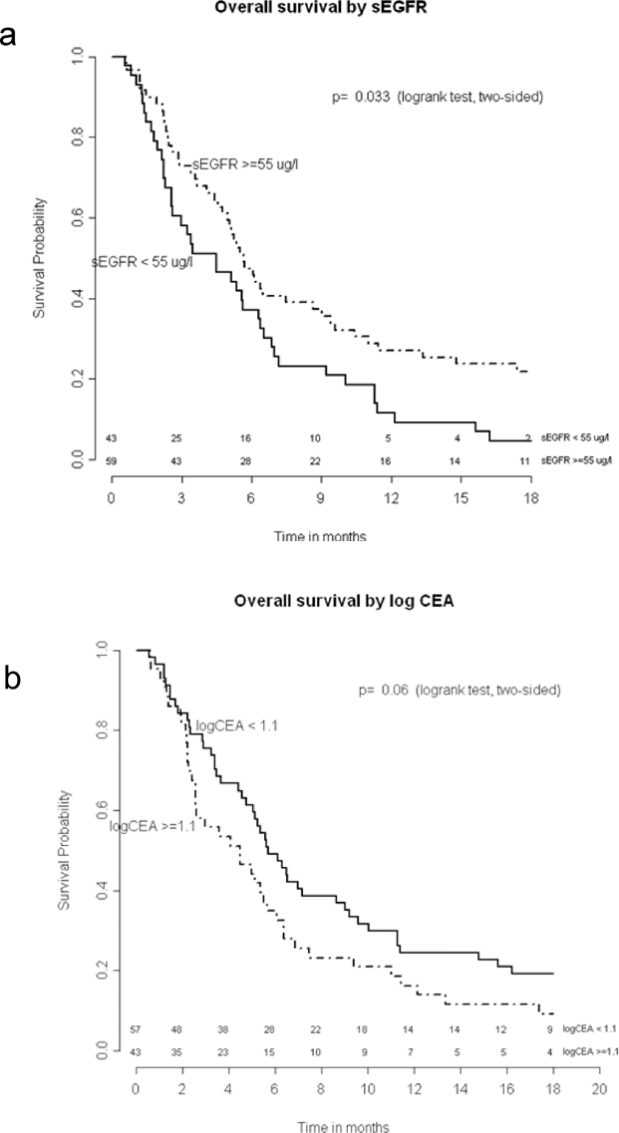
Overall survival by sEGFR and log CEA.

The median overall survival was 5.2 months (range 0.6–52.0 months). In an univariate analysis, smoking status and sEGFR were shown to be significant prognostic factors (Table 2, *p*=0.001 and *p*=0.018, respectively). Cut-off values for sEGFR and log CEA were found at 55 μg/l and 1.1 (corresponding with CEA= 12.6 µg/l), respectively. Patients with sEGFR levels above 55 μg/l had a significantly longer overall survival (Figure 1a, log rank *p*=0.033), while patients with a log CEA level below 1.1 showed a trend toward longer overall survival (Figure 1b, log rank *p*=0.06). In a multivariate overall survival model, sEGFR and log CEA, in addition to smoking, proved to be independently associated with survival (Table 3).

## Discussion

In the present study, baseline sEGFR and CEA levels were measured in patients with advanced NSCLC before the treatment with erlotinib or gefitinib. Our results suggest that higher sEGFR and lower CEA levels are related to prolonged survival in patients receiving EGFR-TKI treatment, indicating that the combination of sEGFR and CEA could be valuable for the selection of patients for the EGFR-TKI treatment.

Carcinoembryonic antigen is a member of the immunoglobulin superfamily and plays a role in cell-to-cell adhesion [35]. When CEA is over-expressed on the cell surface, it is thought to play a role in tumourigenesis by disruption of cell polarity, inhibition of apoptosis (anoikis) and inhibition of cell differentiation [36–38]. The over-expression of CEA has been found to be present in many types of carcinomas [39]. In non-small cell lung cancer, an elevated serum CEA level is generally considered to be a negative prognostic factor especially for adenocarcinoma [40]. Therefore, the finding of Okamoto *et al*. that a high CEA was predictive for good response to the EGFR-TKI treatment, independent of histology, was highly surprising [41]. They did not have an explaining mechanism of action for this phenomenon, but hypothesized that an anti-apoptotic signal of the (mutant) EGFR may somehow elevate the expression level of CEA protein. In this study, we could not confirm the results of Okamoto *et al*. In contrast, we found that a low CEA level was independently associated with better outcome after the treatment with EGFR-TKIs. This is in accordance with the previous findings of the negative prognostic ability of CEA [42]. Our results suggest that serum levels of CEA and sEGFR are not (directly) regulated by the same mechanism of action, since both remained significant upon multivariate analysis. Only few studies are available on sEGFR in non-small cell lung cancer, mostly concerning the comparison of sEGFR levels in healthy individuals and lung cancer patients. Two studies found that patients with NSCLC had lower baseline sEGFR levels compared to healthy controls [43,44], whereas others did not detect significant differences [45,46]. However, up till now, there are no data available on the prognostic value of serum sEGFR for NSCLC. Only one study investigated changes in the sEGFR levels during the EGFR-TKI treatment as a predictive marker for response to these inhibitors [47]. Responders showed a decrease in the sEGFR levels at time of best response compared to baseline level, whereas non-responders showed an increase. A difference of −3.6 μg/l as a cut-off was found to identify responders at time of best response. However, a meaningful cut-off level for (pre-treatment) baseline levels could not be established, and therefore, sEGFR was not considered to be a useful predictive marker. Unfortunately, in our study, serum samples drawn during treatment were not available, and we therefore could not validate these results.

The one-armed design and retrospective nature of our study prohibit clear differentiation between the prognostic and the predictive values of sEGFR and CEA. Interpretation of response data (progressive disease) remains difficult, but these data suggest at least some predictive potential for sEGFR. Higher levels of pre-treatment sEGFR in patients treated with EGFR-TKIs were associated with lower risk of progressive disease within three months.

The prognosis of advanced NSCLC after failure of second or third line treatment is generally only weeks to months. Intensive follow-up with additional imaging during this period is undesirable, and further efforts to evaluate response or progression-free survival are meaningless. The distinction between the prognostic value and the predictive value of these two markers remains important, since their potential predictive value may contribute to an adequate patient selection for expensive EGFR-TKI treatment. Therefore, validation of this potential predictive value in a prospective controlled (two-armed) study is warranted.

In conclusion, these results suggest that sEGFR and CEA are the markers of survival in patients treated with EGFR-TKIs. The potential predictive value of sEGFR needs confirmation in a prospective controlled trial.

## Figures and Tables

**Table 1:**
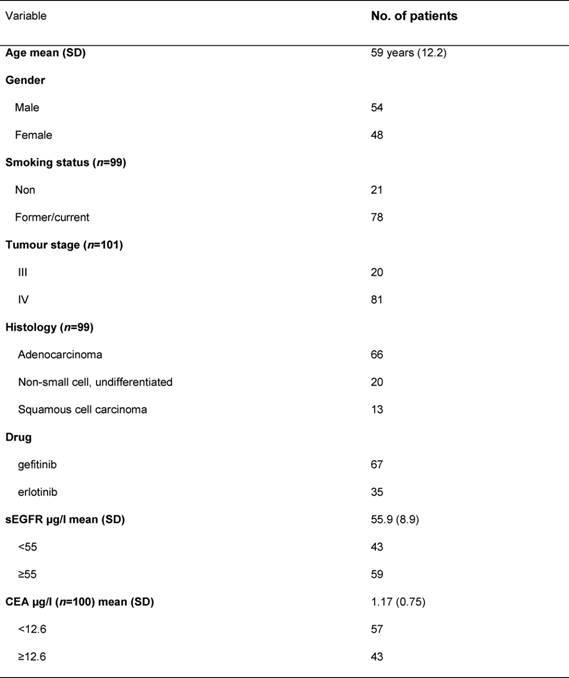
Patient and tumour characteristics (*n*=102)

**Table 2:**
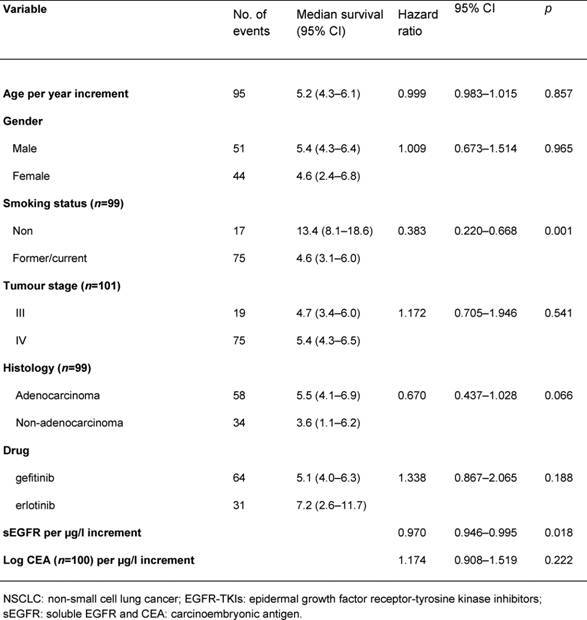
Univariate analysis in patients with advanced NSCLC before treatment with EGFR-TKIs

**Table 3:**
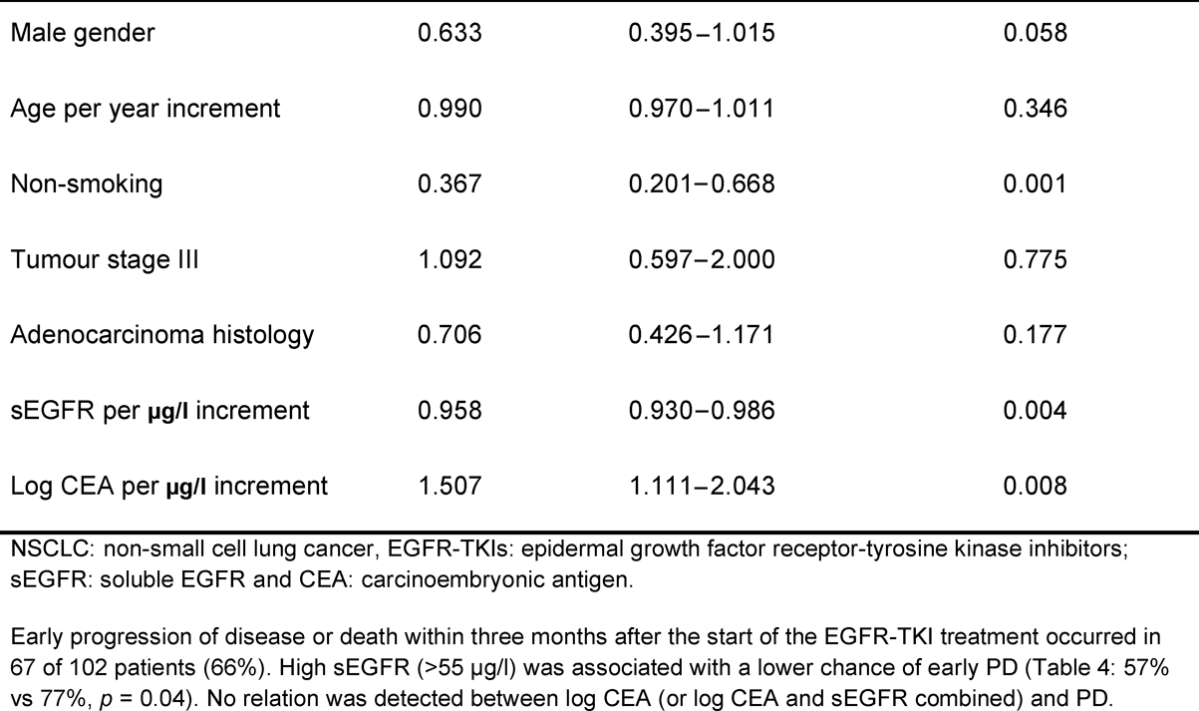
Multivariate overall survival model in patients with advanced NSCLC before treatment with EGFR-TKIs: results of the Cox proportional hazard regression analysis

**Table 4:**
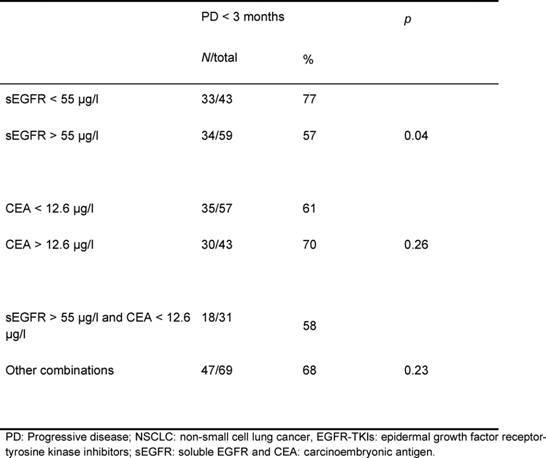
Relation between pre-treatment sEGFR and CEA levels and early progressive disease in patients with advanced NSCLC after treatment with EGFR-TKIs
